# Characterisation of measles after the introduction of the combined measles-mumps-rubella (MMR) vaccine in 2004 with focus on the laboratory data, 2016 to 2019 outbreak, Romania

**DOI:** 10.2807/1560-7917.ES.2019.24.29.1900041

**Published:** 2019-07-18

**Authors:** Mihaela Lazar, Aurora Stănescu, Ana Raquel Penedos, Adriana Pistol

**Affiliations:** 1 Cantacuzino, National Military-Medical Institute for Research and Development, Bucharest, Romania; 2 Research Institute of the University of Bucharest (ICUB), Earth Environmental and Life Sciences Division, Bucharest, Romania; 3 National Centre for Communicable Diseases Surveillance and Control, National Institute of Public Health, Bucharest, Romania; 4 Virus Reference Department, Public Health England, London, United Kingdom

**Keywords:** measles, outbreak, Romania, molecular epidemiology, children, MMR, vaccination, air-borne infections, viral infections, vaccine-preventable diseases, measles-mumps-rubella (MMR) vaccine, laboratory surveillance, epidemiology, laboratory, molecular methods

## Abstract

**Background:**

Since January 2016, a resurgence of measles in Romania has led to the third measles epidemic in the past 12 years; 64 deaths have been confirmed so far–the highest number of measles-related deaths since the measles-mumps-rubella (MMR) vaccine was introduced in 2004.

**Aim:**

To provide an overview on the characterisation on measles in Romania after the introduction of the MMR vaccine with focus on the current outbreak, laboratory and molecular analysis.

**Methods:**

We performed an analysis of measles incidence and mortality after the introduction of MMR vaccination and a retrospective study using serological and molecular data in three consecutive outbreaks with focus on the current outbreak.

**Results:**

In the current outbreak, 17,533 measles cases were notified to the national surveillance system, 93% were unvaccinated. Measles virus was isolated from 429 samples and 283 were genotyped. Genotype B3 was predominant (n = 269) and sporadic measles cases associated with D8 genotype (n = 9) were also observed; genotype D4 and D8 were identified in the previous two measles outbreaks. The detection of several distinct measles virus B3 genotypes suggests multiple virus importations to Romania.

**Conclusion:**

The current outbreak is a consequence of insufficient vaccine coverage. Control measures were implemented to improve uptake of MMR vaccine, including administering the first MMR dose at a younger age (9–11 months) and offering catch-up vaccination to children that have not followed the recommended dosing schedule. More measures are needed to improve the surveillance performance and to achieve high routine MMR vaccination coverage.

## Introduction

Measles is a highly contagious respiratory infection, a viral disease capable of causing epidemics. Unimmunised children and susceptible adults can get severe infection and die from measles due to complications such as pneumonitis and encephalitis. A rare complication of measles is subacute sclerosing panencephalitis (SSPE) which may occur many years after the primary infection, due to persistence of the virus in the central nervous system [1].

In 2012, the World Health Organization (WHO) set a target for measles to be eliminated by 2020 in all WHO Regions. Of 53 countries in the WHO European Region, 43 have interrupted measles endemic transmission as at the end of 2016 [2]. Elimination and maintenance of elimination of endemic measles requires achieving very high levels of population immunity (> 95% vaccination coverage) and good laboratory-based surveillance to rapidly detect and control periodic outbreaks. Remaining pockets of low immunisation coverage allow the virus to spread among those who choose not to vaccinate, or who do not have equitable access to vaccines or cannot be protected through vaccination due to young age or underlying health conditions [3]. In Romania, progress has been made in the reduction of morbidity and mortality from measles since the introduction of a measles vaccine in 1979. However, despite intense effort to eliminate endemic measles, the risk for illness and death from measles is still present as evidenced by the ongoing epidemic in Romania that began in 2016.

### Immunisation history

Prior to the introduction of a measles vaccination policy in May 1979 (imported, live attenuated monovalent measles vaccine), Romania had 60,000–150,000 reported measles cases annually [4]. Between 1979 and 1994, a single dose of measles vaccine was administered to children aged 12–15 months. The vaccine was produced by the National Institute Cantacuzino in Bucharest [5]. In 1994, a second dose of measles vaccine was introduced for children entering school at age 7 years. Since the introduction of vaccination, annual cases have remained below 5,000 – except during large outbreaks, when 30,000–60,000 cases were reported [6]. A mass measles and rubella immunisation campaign, in which 2.1 million children were vaccinated, was conducted following an outbreak in 1996–98 that led to over 33,000 cases and 21 deaths. In this outbreak, the majority of cases occurred in unvaccinated children aged less than 2 years and children of school age (5-7 years old). The measles-mumps-rubella (MMR) vaccine replaced the monovalent measles vaccine in 2004 and was recommended for children aged 12 to 15 months. The second MMR vaccine dose was introduced in October 2005 for children aged 6–7 years. From 2005 to 2009, reported first dose coverage with the MMR vaccine was over 95% [7]. In 2015, the coverage had decreased to 85.8% (dose 1; 12–15 months) and 67% (dose 2; 7 years). Supplementary vaccination campaigns led to an increase in MMR vaccine coverage to 87% (dose 1) and 75% (dose 2) in 2017 [7,8]. The vaccination coverage from the official Romanian statistics refers only to people included in the healthcare system, with birth certificates and medical records. There are still unregistered children in the system, especially among communities that are marginalised or affected by extreme poverty. More attention is needed to target this group, but the size of the underreported population is not known [9,10].

### Measles incidence since 1960

**Figure 1:**
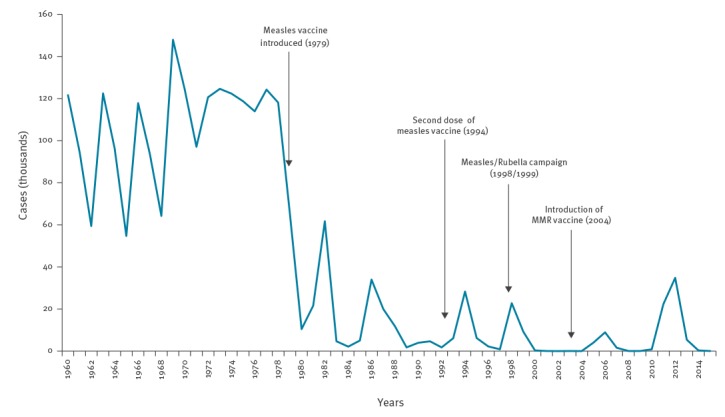
Incidence of measles in Romania,1960–2015

Following the introduction of measles vaccination in 1979, measles epidemics have occurred in 1982, 1986, 1993, 1997, 2003, 2010 and 2016 in Romania, but number of cases and measles-related deaths is much less in comparison to epidemics in the pre-vaccination era (Figure 1).

MMR: measles-mumps-rubella.

Information about the genotype and virus variant (named strain) is becoming increasingly important to discriminate between endemic and imported virus variants, wild-type and vaccine strains (in recently vaccinated persons) and to track transmission routes as part of routine surveillance [11]. Here, we describe the measles situation in Romania after the introduction of the combined MMR vaccine in 2004, with particular focus on the 2016–19 outbreak, and the demographic characteristics of reported measles cases and the molecular epidemiology of circulating measles strains.

## Methods

### Case definition

Measles is a notifiable disease in Romania requiring physicians and laboratories to immediately notify local public health units of all probable and confirmed cases, according to the national strategy of measles surveillance, approved by Romanian Ministry of Health. Case-based surveillance of measles is conducted continuously and based on the European Union case definition [12].

Box **Definition of measles cases in Romania**

**Possible case:** A person with fever and maculopapular rash and at least one of the following symptoms: cough, coryza, or conjunctivitis

**Confirmed case:** A case that was not recently vaccinated and meets the clinical definition and the laboratory criteria:

- Detecting measles IgM antibody in serum samples; or

- Isolation of measles virus from a clinical specimen; or

- Detection of measles-virus specific nucleic acid from a clinical specimen using PCR; or

- IgG seroconversion or a significant rise in measles IgG antibody (validated method)

**Probable case:** A case who met the clinical case definition with an epidemiological link to a laboratory-confirmed case (contact with a confirmed measles case).

### Collection and processing of samples

Blood samples were collected from possible measles cases and tested in the National Measles and Rubella Reference Laboratory in Romania based in Cantacuzino National Military Medical Institute for Research and Development, Regional Laboratories, Iasi, Cluj, Timisoara and Bucharest and in laboratories of infectious diseases hospitals around Romania. All laboratory-confirmed cases are reported to the surveillance system located at National Institute of Public Health, National Centre for Communicable Diseases Surveillance and Control (Bucharest).

Throat swabs collected in viral transport medium (VTM), urine, necropsy samples and cerebrospinal fluid (CSF) were transferred for virological testing in Cantacuzino Institute laboratory.

### Laboratory data

Detection of specific anti-measles virus IgM antibodies by enzyme-linked immunosorbent assay (ELISA) in serum is the reference standard for the confirmation of measles. Serum samples were tested using Measles virus IgM micro-capture ELISA (IBL International GMBH, Hamburg, Germany) or Enzygnost Anti-Measles Virus/IgM antibody enzyme immunoassay (EIA; Siemens, Marburg, Germany) according to the manufacturer’s instructions. All serum samples with negative results were tested for the rubella-specific IgM using Rubella virus IgM micro-capture ELISA (IBL International GMBH, Hamburg, Germany). Measles infection was confirmed when anti-measles IgM antibodies were present. In the event of an equivocal result, a second serum or swab/oral fluid was requested to ascertain seroconversion or to identify the measles virus.

Clinical specimens such as swabs, urine, cerebrospinal fluid (CSF), saliva and lung fragments were used for viral RNA extraction directly from 150 μl of sample using the Nucleospin Viral RNA kit (Macherey, Germany) according to the manufacturer’s instructions. Each RNA sample was stored at − 70 °C until amplification by real-time reverse transcription PCR (qRT-PCR). Sequencing of the 450 nt of the C-terminal region of the measles virus nucleoprotein gene (N-450) was attempted to determine the genotype by using the one-step RT-PCR kit according to manufacturer's protocol (QIAGEN OneStep RT-PCR Kit, Hilden, Germany) [13].

Confirmed measles cases from new small (< 10 cases) outbreaks (index case and two or three secondary cases) with a history of travel abroad during the incubation period (7–21 days) were selected for genotyping; severe cases were also genotyped. To sequence the DNA templates, the PRISM BigDye Terminator v3.1 Ready Reaction Cycle Sequencing kit (Applied Biosystems, Foster City, California, United States (US)) was used on a PRISM 3100-Avant Genetic Analyzer (Applied Biosystems).

### Sequence analysis

N-450 sequences were aligned against related sequences retrieved from GenBank database using the BioEdit Sequence alignment Editor. MEGA-X software was used for constructing a Maximum Likelihood Tree using the Tamura-Nei evolutionary model with neighbour-joining using 100 bootstrap replicates. The phylogenetic relationship between virus variants was taken into account when transmission chains were analysed in the European context. The sequences reported in this study have been deposited into the Measles Nt Surveillance database (MeaNS), a tool used to track measles sequence diversity and monitor elimination of virus strains [14] and GenBank database under accession numbers (KX372735-KX372737, MX671734-MK671996) [15]. The nomenclature followed WHO recommendations [16].

## Results

Overall, between 1 January 2016 and 1 July 2019, 17,533 cases of measles were notified to the national surveillance system corresponding to an annual incidence of 122.07 per one million population in 2016 increasing to 459.4/million in 2017. From the notified cases, 9,673 were laboratory-confirmed cases and 7,860 were probable cases.

### Characteristics of cases and vaccination status

**Figure 2:**
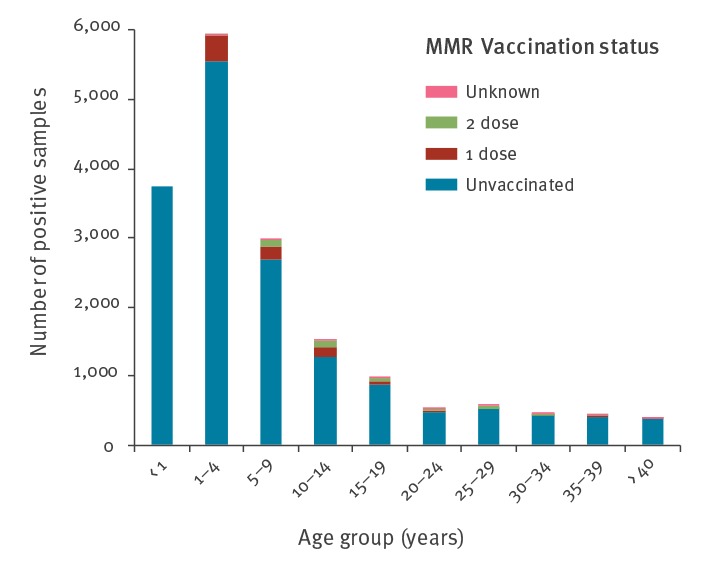
Measles cases by vaccination status, Romania, January 2016–July 2019 (n = 7,533)

Of the 17,533 cases reported, 77% (n = 13,424) were among individuals who were eligible for vaccination but were incompletely vaccinated and 23% (n = 4,109) had an immunisation status considered ‘up to date for age’ with the vast majority being infants < 1 year (n = 3,739; 91%) i.e. they were too young to receive their first dose of MMR vaccine or they were adults born before 1979 when the measles-containing vaccine was introduced (n = 371). Infants and young children aged 9 years or less were the most affected age group (n = 11,977). Cases were reported in patients aged 3 weeks–59 years (median age: 9 years) (Figure 2).

MMR: measles-mumps-rubella.

**Figure 3:**
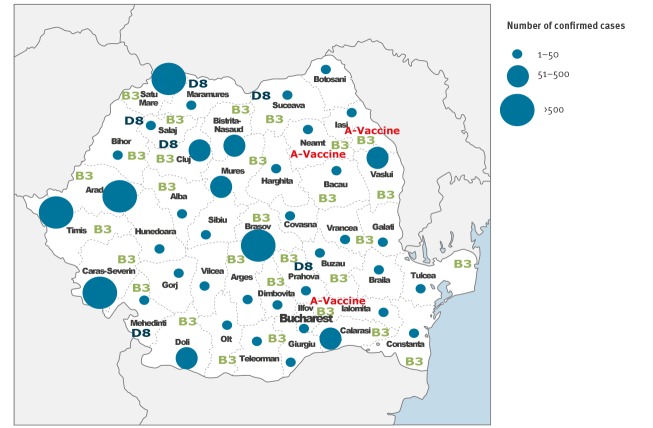
Geographical distribution of serologically confirmed measles cases and virus genotype, Romania, January 2016–January 2019 (n = 9,673)

Cases have been reported in all 42 districts of Romania with the districts of Timis (n = 1,228), Caras Severin (n = 1,112), Arad (n = 1,014), Satu Mare (n = 951) and Brasov (n = 867), situated in the west and centre of Romania, reported the highest number of cases (Figure 3).

### Mortality

The 2004–07 measles outbreak resulted in 23 fatalities; four cases were registered in 2010–13 (National Institute of Public Health, data not shown). Measles-related deaths were recorded in 64 cases in the 2016–19 outbreak (60 laboratory-confirmed cases and four were epidemiologically linked), corresponding to a case fatality ratio (CFR) of 0.4 per 100 measles cases. The deaths occurred as a consequence of severe measles complications; 41 cases (93.3%) had acute pneumonia and four cases (6.7%) suffered acute encephalitis. Fourty cases had a pre-existing medical condition including: anaemia (n = 8), malnutrition (n = 7), cardiac diseases (n = 4), spastic tetra-paresis (n = 4), immunosuppressed (n = 3), born prematurely (n = 2), Down syndrome (n = 1), Niemann Pick disease (n = 1), cystic fibrosis (n = 1), seizure syndrome (n = 1), granulomatous disease (n = 1), pulmonary tuberculosis under treatment (n = 1), intellectual disability (n=2, one case was confirmed for measles and influenza), but 24 cases recorded no pre-existing medical condition.

All but one fatal case occurred in unvaccinated patients who were aged 1 year or less (n = 45); the exception had hypotrophy and anaemia (aged 4 years) and had been vaccinated with one dose of MMR as per the national scheme.

The median age at death was 8 months (range: 21 days–41 years). The most affected counties were Timis (n = 9), Dolj (n = 7), Arad (n = 6), Constanta (n = 5), Iasi (n= 5), Bacau (n = 4), Neamt (n = 4) and Caras Severin (n = 3).

### Laboratory confirmation

From January 2016 to July 2019, 11,713 serum samples collected from possible measles cases were tested, 9,596 (82.5%) of which were IgM positive, 67 with equivocal results (0.6%). Of cases with IgM equivocal results, swab samples were available from 59 all of which were positive for measles virus. A second serum sample was requested from eight cases and their ELISA test results were positive for specific IgM.

All 2,040 (16.9%) negative serum samples were tested for the presence of IgM specific for rubella, none of them was positive. All serum samples collected from possible rubella cases (n = 169) were tested for measles IgM, 10 were positive. The initially laboratory-confirmed cases reported no history of travel abroad.

**Figure 4:**
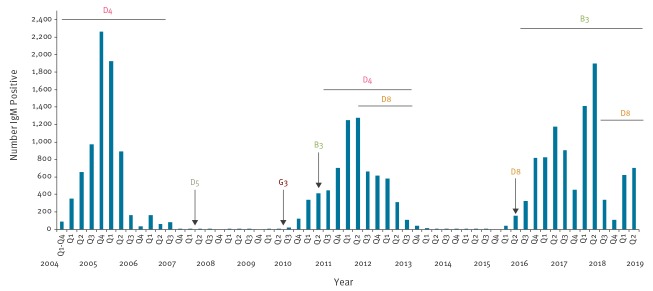
Quarterly distribution of confirmed measles cases and genotypes, Romania, 2004–2018 (n = 24,399)

For the viral detection, 578 specimens were collected within 6 days of rash onset. The viral RNA was extracted directly from 429 samples (403 swabs, 16 lung fragments, 5 serum, 2 urine samples, 2 CSF and 1 saliva). The rest of 149 specimens were negative for the presence of measles virus. The genotype was determined for 283 selected samples (index case and two or three secondary cases from new outbreaks and all cases with a history of travel abroad during the incubation period) of which 278 showed a wild-type virus with genotype B3 (n = 269) and D8 (n = 9). Five sequences matched a vaccine strain (A-vaccine), being associated with reactions to recent vaccination rather than measles cases. The serological and molecular results were compared with the laboratory results from the previous two measles epidemics after the introduction of MMR vaccination in Romania in 2004 (Figure 4).

### Measles Virus Genotype

The two measles outbreaks 2004–07 and 2010–13 were mostly caused by measles strains of genotype D4. In 2004, the outbreak was associated with the strains D4-Bucharest and D4-Hamburg. The MV variant D4-Manchester and sub-variant MVs/Maramures.ROU/3.11 caused prolonged outbreaks associated with secondary spread that resulted in additional outbreaks in 2010–13 (data not shown).

### Phylogenetic analysis of measles virus B3 Genotype

Between January 2016 and July 2019, 95% (n = 269) of the sequenced samples belonged to genotype B3.

**Figure 5:**
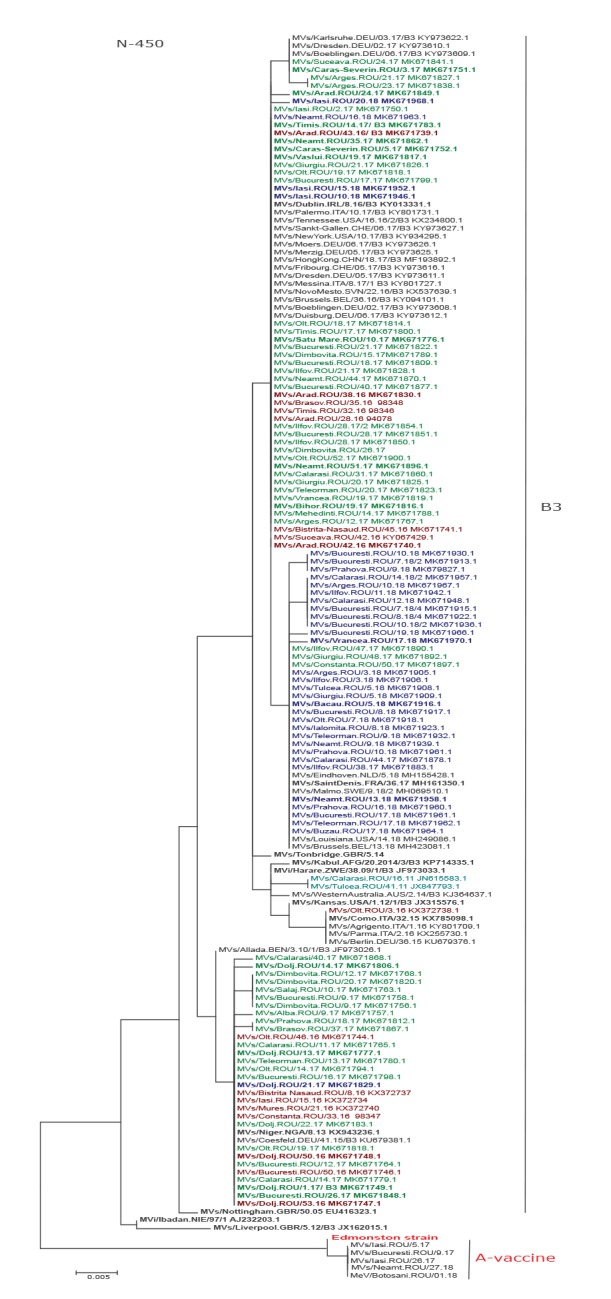
Phylogenetic relationship between representative measles B3 strains, Romania

Phylogenetic analysis of these strains showed that the sequences were divergent, with distinct lineages within Romania being identifiable. The first sporadic case identified in the most recent outbreak was detected in January 2016 from a patient with no history of travel aboard (MVs/Olt.ROU/3.16). The sequence is 100% identical to the named strain MVs/Como.ITA/32.15 and sequences from Berlin (Germany) and Agrigendo, Parma (Italy) (Figure 5) and did not appear to spread further.

Measles strains: 2016 is shown in burgundy, 2017 in green and 2018 in dark blue. Sequences from decreased people are in bold. All sequences from 2011 are shown in light blue and sequences non-Romanian publically available in relation to named strains in black and bold.

Phylogenetic analysis was performed using MEGA-X software with a neighbour-joining (NJ) algorithm inferred with Tamura-Nei parameter for sequence evolution.

A Romanian measles lineage has 42 sequences with 100% identity to the named strain MVs/Niger.NGA/8.13 and a sequence from Coesfeld, Germany (MVs/Coesfeld.DEU/41.15; KU679381.1). The first sequence from this lineage was obtained in Bistrita Nasaud County in February 2016 (MVs/Bistrita-Nasaud.ROU/8.16) and initiated a small outbreak in the region with 40 cases. The same virus seeded outbreaks in other counties i.e. Iasi, Mures, Constanta, Dolj, Olt, Bucuresti, Calarasi and Teleorman. Variants divergent by one nt were detected from sporadic cases: MVs/Alba.ROU/9.17, MVs/Dolj.ROU/14.17, MVs/Prahova.ROU/19.17 and MVs/Brasov.ROU/37.17. Seventeen identical sequences divergent by one nt evolved into a different lineage from MVs/Niger.NGA/8.13 and were linked to measles outbreaks in Dambovita county and Bucuresti.

Another lineage with 93 sequences identical to the named strain MVs/Dublin.IRL/8.16 (KY013331.1) and sequences from Belarus, China, Germany, Italy, Serbia and the US was identified. Some cases were divergent from this lineage by one nt: MVs/Arad.ROU/24.17, MVs/Cluj.ROU/28.17 and MVs/Iasi.ROU/20.18. The strains responsible for outbreaks in the counties of Arges (MVs/Arges.ROU/21.17) and Dolj (MVs/Dolj.ROU/23.17) diverged from the Dublin named strain by two nt. Two sporadic cases with identical N-450 sequences divergent from this third lineage by one nt were also identified in week 3 and 24 in 2017 (MVs/Caras-Severin.ROU/3.17, MVs/Suceava.ROU/24.17). Identical sequences were also found in Germany (MVs/Dresden.DEU/02.17, MVs/Karlsruhe.DEU/03.17, MVs/Boeblingen.DEU/06.17).

The third lineage contains 83 sequences identical with named strain MVs/Saint-Denis.FRA/36.17 and sequences from Belgium, the Netherlands, Sweden and the US, (MVs/Brussels.BEL/13.18 MVs/Eindhoven.NLD/12.18, MVs/Malmo.SWE/9.18/2 and MVs/Louisiana.US/14.18). Variants divergent from this strain by a single nt were identified in Vrancea (MVs/Vrancea.ROU/17.18), Prahova (MVs/Prahova.ROU/16.18), Calarasi (MVs/Calarasi.ROU/12.18), Arges (MVs/Arges.ROU/10.18), Ilfov (MVs/Ilfov.ROU/11.18) counties and Bucuresti (MVs/Bucuresti.ROU/19.18) (Figure 5).

A sporadic case with measles genotype B3 was also detected in April 2011 in Calarasi County (MVs/Calarasi.ROU/16.11), the case had travelled to Spain. Phylogenetic analysis of this case revealed 100% identity with MVs/Barcelona.ESP/48.10 detected between 2010 and 2011 in Spain. Another case with an identical sequence (MVs/Tulcea.ROU/41.11) was detected 25 weeks later in the neighbouring Tulcea County and apparently did not spread further (Figure 5).

### Phylogenetic Analysis of measles virus D8 Genotype

**Figure 6:**
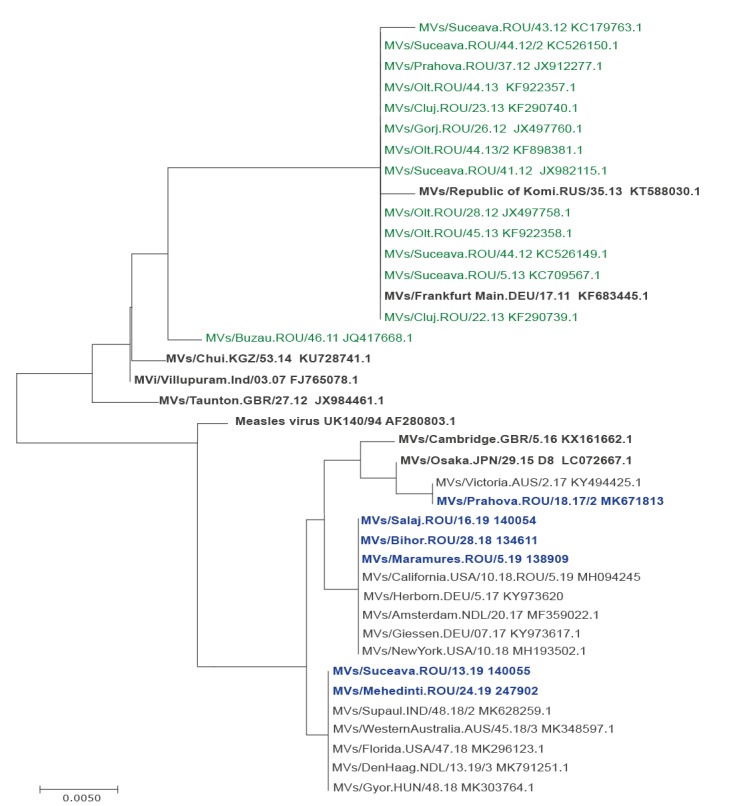
Phylogenetic tree of 18 cases infected with measles virus genotype D8, Romania, 2012–2019

Nine of 283 samples collected in the period January 2016–July 2019 belonged to measles genotype D8. The first patient infected with this virus (MVs/Prahova.ROU/18.17/2) had a history of travel to Germany during the incubation period (7-21 days). The transmission pathway could not be traced. Basic Local Alignment Search Tool analysis of the N-450 sequence showed that it was 100% identical to a sequence from Australia (MVs/Victoria.AUS/2.17, KY494425). Another two sequences with the same genotype were identified at patients from Bihor County with a history travel in France (MVs/Bihor.ROU/28.18); identical sequences were identified in week 5 (MVs/Maramures.ROU/5.19) and week 16 (MVs/Salaj.ROU/16.19) in 2019. Other four sequences different by one nucleotide were identified in Suceava and Mehedinti Counties (MVs/Suceava.ROU/13.19, MVs/Mehedinti.ROU/24.19), 100% identical with sequences from Amsterdam, Hungary, India, Nederland and US. The phylogenetic analysis showed that the sequences are identical with other public sequences from Germany, Netherland and the US. The genotype D8 was also detected in Romania in November 2011 in Buzau County (MVs/Buzau.ROU/46.11), but no secondary cases were detected. Other measles cases associated with this genotype were detected in 2012 in Gorj, Olt and Suceava counties and included a fatal case: MVs/Suceava.ROU/41.12. These strains belonged to the variant D8-Frankfurt-Main (MVs/Frankfurt Main.DEU/17.11) (Figure 6).

Sequences from the 2016–19 are marked in blue and from the 2011–12 in green, in relation to named strains (black bolded) and other public sequences.

Phylogenetic analysis was performed using MEGA-X software with a neighbour-joining (NJ) algorithm inferred with Tamura-Nei parameter for sequence evolution.

## Discussion

Since 2016, Romania has been facing a measles outbreak with three distinct lineages of B3 measles genotype and sporadic D8 genotype being identified. No index case was identified. The molecular epidemiology suggests that there are different chains of transmission. The outbreak occurred primarily among unvaccinated or partially vaccinated individuals (95% and 3.8% of cases, respectively); only 1.2% of cases were in individuals who had received two doses of MMR vaccine and no deaths were reported in the latter group. Children have been most affected by the outbreak, with more than 80% of cases being aged 15 years or less.

We showed that despite implemented and ongoing control measures on a national level e.g. lowering the age of first dose of MMR to 9–11 months of age and supplementary vaccination activities, Romania still ranks high among countries with endemic transmission of measles infection. Since the introduction of the MMR vaccine in 2004, epidemics of measles have still occurred every 2–4 years and while in the current outbreak the western part of Romania has been most affected, in the measles outbreak 2010–13 the eastern region was most affected [10].

Changes in the main circulating strains from D4 to D8 and now B3, reflect the reported genotypes in Europe [14,17-23]. The present outbreak raises concern due to the large number of deaths (n = 64) and the higher infant mortality compared with the previous ones [10]. The majority of measles-related deaths were caused by complications associated with the disease in very young children (up to 12 months of age) whose immune systems are not fully developed and less able to fight the virus. More studies are needed in order to identify the cause of the higher mortality compared with the past measles outbreaks.

To reduce mortality from measles, several strategies have been implemented relating to the vaccination programme in Romania. However, the vaccination coverage remains under 90% allowing for continued measles transmission within the country and the potential for spread to other countries with suboptimal vaccination coverage. A survey of parents conducted in 2015 found that the most common reasons for them not to vaccinate their children were failure to attend a healthcare clinic and refusal of vaccines based on safety concerns – despite the historic and strong evidence of safety and efficacy of vaccines against measles [24,25]. The Romanian government plans to accelerate the response to the measles outbreak through: (i) far-reaching, countrywide vaccination campaigns, to establish an effective vaccine management system (ii) amending legislation regarding the purchasing of vaccines to keep the process transparent and predictable) development of a multi-year plan for assessing the need for vaccines, and (iv) build a national stock of vaccines for exceptional situations (e.g. outbreaks). The National Centre for Communicable Diseases Surveillance and Control publishes weekly measles bulletins on the websites of the national institute of public health to provide timely information about the measles situation [26]. In Romania, there are protocols to guide surveillance, outbreak investigation and response but there is a need to dedicate additional resources for molecular surveillance (resources for staffing, laboratory support, training and other operational costs).

Measles transmission is difficult to assess through epidemiological information alone as on average, one third of cases have no identifiable link to another case [27]. PCR-based testing is able to diagnose measles with recent symptom onset while serology can be used for patients who are later in their clinical course (or for retrospective case identification), as measles IgM typically develops up to 1 week after rash onset [28]. During outbreaks, it is important to be able to rapidly distinguish between measles cases and vaccine reactions to avoid unnecessary outbreak response measures e.g. case isolation and contact investigations [29]. More information on strain sequences, for example, could provide valuable information that can contribute to the epidemiological investigation [30]. Genetic characterisation of measles virus has been crucial to understanding both the biology and evolution of measles viruses and has become an integral part of routine laboratory surveillance. Sequencing of additional genes could confirm a line of descent that is otherwise only ‘presumed’ on the basis of the sequence minimum routinely determined for genotyping.

### Conclusions

During the ongoing outbreak in Romania, the measles has resulted in a higher number of deaths than any other vaccine-preventable disease among children. The suboptimal MMR coverage rate has led to the accumulation of susceptible individuals. The use of timely genotyping, epidemiological field investigation (linked with laboratory analyses) to determine the source of infection could lead to a better understanding of the gaps in national surveillance, the root cause(s) of measles outbreaks, how future outbreaks can be prevented in order to reach the goal of measles elimination. In the absence of a travel history or an epidemiological link between most cases, extended genotyping could be used to seek additional evidence about possible chains of transmission to improve measles surveillance.
